# *Streptococcus intermedius *causing infective endocarditis and abscesses: a report of three cases and review of the literature

**DOI:** 10.1186/1471-2334-8-154

**Published:** 2008-11-10

**Authors:** MaryAnn P Tran, Molly Caldwell-McMillan, Walid Khalife, Vincent B Young

**Affiliations:** 1 Department of Medicine, Infectious Diseases Division, Michigan State University, East Lansing, Michigan, USA; 2 Department of Medicine, Michigan State University, East Lansing, Michigan, USA; 3 Department of Microbiology, Sparrow Health Systems, Lansing, Michigan, USA; 4 Department of Microbiology & Molecular Genetics, Michigan State University, East Lansing, Michigan, USA; 5 National Food Safety and Toxicology Center, Michigan State University, East Lansing, Michigan, USA

## Abstract

**Background:**

*Streptococcus intermedius *is a member of the Streptococcus anginosus group. Clinical disease with *S. intermedius *is characterized by abscess formation and rarely endocarditis. Identification of *Streptococcus intermedius *is difficult, leading to the development of molecular methods to more accurately identify and characterize this organism.

**Case presentation:**

Over a period of 6 months we encountered three cases of invasive *Streptococcus intermedius *infection presenting as hepatic abscesses, brain abscess, and endocarditis. We confirmed our microbiologic diagnosis through 16S sequencing and found a common virulence gene in each case.

**Conclusion:**

Our report illustrates three different clinical manifestations due to *Streptococcus intermedius *infection that can be encountered in healthy individuals in a community hospital setting. To our knowledge, this is the first case of *Streptococcus intermedius *endocarditis confirmed by 16S sequencing analysis. The use of molecular methods may allow a better understanding of the epidemiology and pathogenesis of this organism.

## Background

*Streptococcus intermedius *is a member of the Streptococcus anginosus group (SAG), also known as the "*Streptococcus milleri*" group. A variety of clinical diseases have been associated with infection with the different members of the SAG [1]. *S. intermedius *has a tendency to cause abscess formation commonly found in the liver and brain, but is rarely the etiologic agent in infective endocarditis [2-4]. Furthermore, members of the SAG are difficult to distinguish on the basis of phenotypic characteristics [1]. This prompted the development of genotypic methods employing the polymerase chain reaction (PCR) to aid in the speciation of these organisms. We report three varied cases of infection caused by *S. intermedius *that were encountered over a short time period in a community-based teaching hospital. Two cases presented with multiple liver abscesses and bacteremia caused by *S. intermedius*, with one case complicated by the development of endocarditis. The third patient presented with a brain abscess secondary to chronic sinusitis.

## Case presentation #1

A 24 year-old male presented to his primary care physician with an 8-day history of fevers, chills, and myalgias. He complained of a sore throat and worsening headache with neck pain for the past 2 days. He also had abdominal pain, constipation, and bloating throughout the week, without nausea or vomiting. At his primary care physician's office, he had a temperature of 104°F and was sent to the emergency department for further evaluation. On admission, the patient was awake and oriented, but appeared lethargic and pale. Vital signs included a temperature of 103°F, blood pressure of 112/51 mmHg, pulse of 112/minute, respiratory rate of 22/minute, and an oxygen saturation of 88% on room air. The physical exam revealed minimal crackles at the lung bases and mid-abdominal tenderness without peritoneal signs or hepatosplenomegaly. The rest of the physical exam was unremarkable. Laboratory data included a white blood count of 14,900/μL with 77% neutrophils and 10% bands, hemoglobin of 14.9 gm/dL, platelet count of 244,000/cmm, and a C-reactive protein of 39.9 mg/L. Liver enzymes, BUN, creatinine, and electrolyte panel were within normal limits. A monospot and influenza A and B were negative. A lumber puncture performed in the emergency department did not show evidence of meningitis. A chest x-ray showed a left basilar infiltrate.

**Figure 1:**
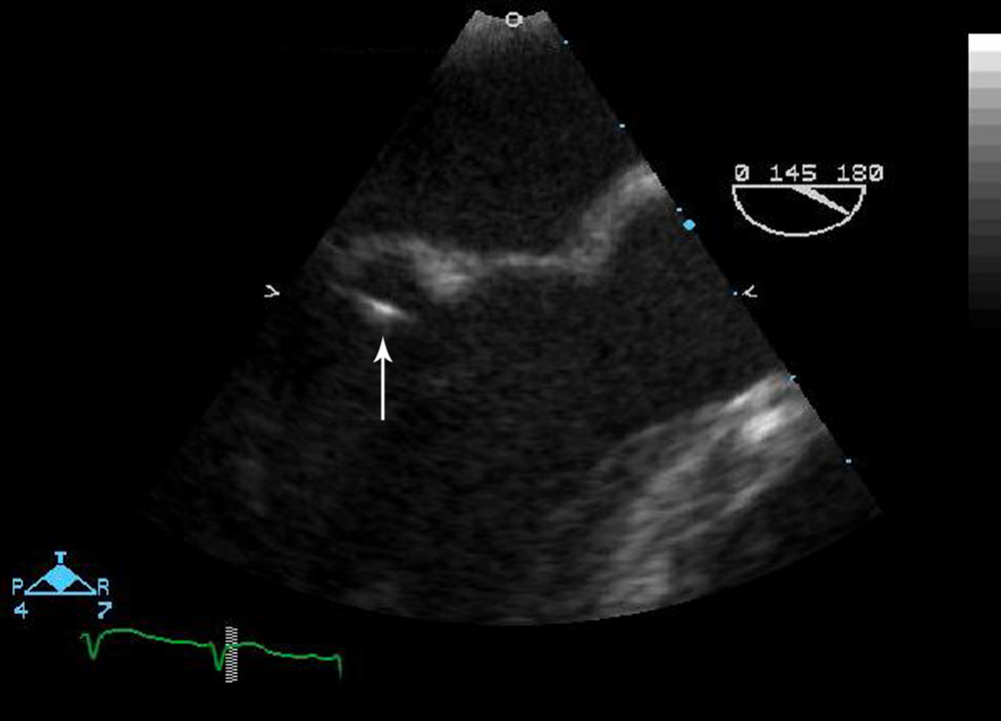
Transesophageal echocardiogram image of the mitral valve showing a highly mobile linear echodensity on the ventricular side of the mitral leaflet.

The patient was initially treated for community-acquired pneumonia with ceftriaxone 2 g once daily and azithromycin 500 mg once daily. Blood cultures drawn on admission grew viridans group streptococci, and azithromycin was discontinued. A transesophageal echocardiogram showed vegetation on the anterior leaflet of the mitral valve (Figure 1), and ceftriaxone was continued for treatment of endocarditis. A CT scan of the abdomen performed due to persistent abdominal pain revealed multiple liver abscesses. One of the abscesses was drained percutaneously under CT guidance. Cultures of the aspirate also grew only viridans group streptococci. Anaerobic cultures were negative. The patient continued to spike fevers, and antibiotics were changed to penicillin 24 million units a day and low dose gentamicin. Repeat blood cultures were negative. However, the patient remained febrile and tachycardic with increasing leukocytosis. Because of worsening clinical status, he required additional percutaneous drainage of three more abscesses. After six months of antibiotic therapy, the patient is doing well with significant clinical improvement.

## Case presentation #2

**Figure 2:**
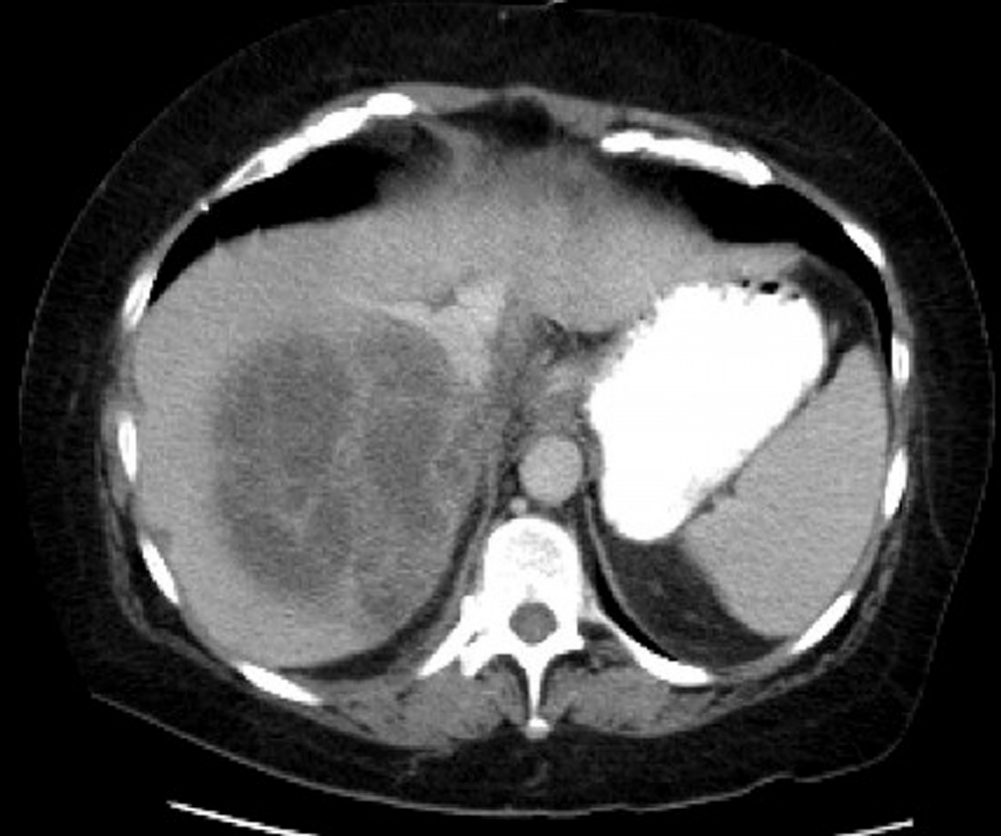
CT scan of the abdomen with contrast of Case 2 showing a large, loculated liver abscess measuring 10 cm.

A 53 year-old female presented to the emergency department with fevers and chills for 12 days. Two days prior to admission, she had nausea, vomiting, and diarrhea. She denied abdominal pain, hematemesis, or hematochezia. She reported a 30 lb weight loss in the past six months, which she attributed to her new job. She drank 1–2 mixed drinks of vodka a day but denied problems with alcohol intoxication or withdrawal. On admission, the patient was alert and oriented, but appeared ill. Vital signs revealed a temperature of 102.5°F, blood pressure of 115/65, pulse 78/minute, and respiratory rate of 20/minute. Physical exam demonstrated right upper quadrant tenderness without distention, guarding, or rebound. The rest of her physical exam was unremarkable. Laboratory data showed a white blood count of 16,000/μL with 89% neutrophils and 1% bands, hemoglobin of 11.9 g/dL, platelets of 361,000/L, alkaline phosphatase of 220 units/L, bilirubin of 2.5 mg/dL, ALT of 51 units/L, and AST of 56 units/L. Amylase, lipase, electrolyte panel, BUN, and creatinine were within normal limits. A CT scan of the abdomen and pelvis showed multiple liver lesions with the largest measuring 10 cm by 9.9 cm with gallbladder wall thickening (Figure 2). An echocardiogram was not performed.

The patient was started on imipenem 500 mg every 8 hours. General surgery was consulted and performed an exploratory laparotomy with surgical drainage of 4 abscesses and a cholecystectomy. Blood cultures on admission grew viridans group streptococci. Surgical cultures also grew viridans group streptococci with negative anaerobic cultures. The antibiotic regimen was changed to ceftriaxone 2 g once daily. The patient continued to follow up with the Infectious Diseases Clinic. After a total of 6 months of cephalosporin therapy, repeat CT scan of the abdomen showed resolution of the liver abscesses.

## Case presentation #3

A 16 year-old female with a history of chronic sinusitis presented with worsening frontal headache and frequent episodes of bloody sinus drainage over a period of six months. She was placed on antihistamines and steroid nasal spray without relief. The patient had difficulty concentrating in school and was started on atomoxetine for possible ADHD. She denied fevers, decreased level of consciousness, and focal neurological signs. An outpatient CT of the brain showed a left frontal mass, and complete opacification of the bilateral frontal, bilateral ethmoid, left maxillary, and right sphenoid sinuses. An MRI was performed which showed a thick-walled, ring-enhancing, frontal abscess, reflecting as an intracranial extension of a large mucocele. She was subsequently sent to the emergency department for further evaluation. Vital signs include a temperature of 97°F, blood pressure of 117/75, pulse 84/minute, and respiratory rate of 14/minute. Physical exam was unremarkable with a GCS of 15 and no neurological deficits. Laboratory data showed a white blood count of 8,100/μL, hemoglobin of 12.5 g/dL, and platelets of 435,000/L. Electrolyte panel, liver function tests, BUN, and creatinine were within normal limits. She was placed on ceftriaxone 2 g every 12 hours and metronidazole 500 mg every 6 hours.

**Figure 3:**
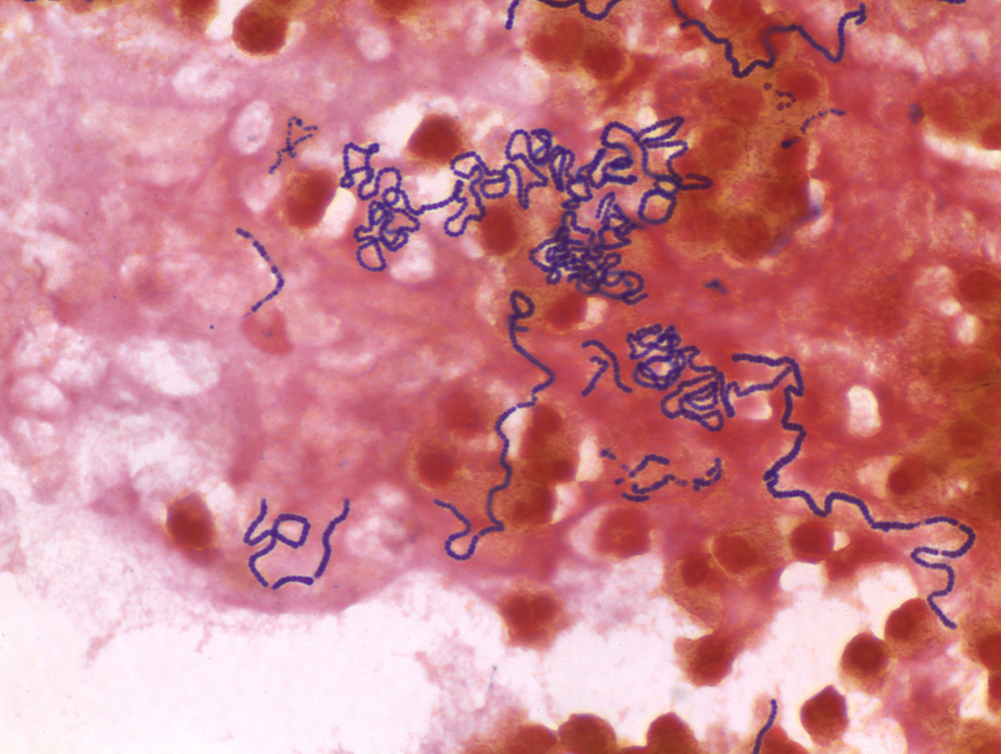
Gram stain of brain tissue from Case 3 showing gram positive cocci in chains.

On the following day, the patient had a bifrontal craniotomy with evacuation of the abscess and cranialization of the frontal sinuses. Pathology of the surgical specimen revealed abscess and granulation tissue with acute and chronic inflammation. A gram stain of the brain tissue showed gram positive cocci in chains (Figure 3). Cultures from the surgical drainage grew viridans group streptococci with negative anaerobic cultures. Because of the extent and severity of the sinusitis, the patient underwent bilateral ethmoidectomy, bilateral maxillary antrostomy, left sphenoidotomy, and left concha bullosa resection. The patient finished an 8-week course of ceftriaxone monotherapy. A repeat MRI of the brain showed resolution of the frontal abscess.

## Microbiologic diagnosis

Written consent was obtained from all patients. Michigan State University's Institutional Review Board has waived the need for approval.

Direct Gram stain of the abscess drainage of all three patients and positive blood cultures revealed Gram-positive cocci in chains. Cultures on sheep blood agar produced alpha-hemolytic colonies of Gram-positive cocci after 24 hours of incubation at 37°C. The isolates were presumptively identified as members of the Streptococcus anginosus group by phenotypic classification [5]. Antibiotic susceptibility testing was performed by E-test on both blood and abscess drainage cultures. The isolate of case #1 was susceptible to penicillin (MIC ≤ 0.12 mg/L), ceftriaxone (MIC ≤ 0.12), cefotaxime (MIC ≤ 1.25), and vancomycin. The isolate was resistant to erythromycin and clindamycin. The blood and surgical abscess drainage cultures of case #2 revealed similar susceptibility patterns in that the isolate was susceptible to penicillin (MIC ≤ 0.032), ceftriaxone (MIC ≤ 0.064), and vancomycin with resistance to erythromycin and clindamycin. Tetracycline was not tested in both cases. The isolate from the brain abscess of case #3 did not have adequate growth to perform antibiotic susceptibilities.

Using the primer pair 8F and 1492R and the genomic DNA from each isolate, a nearly complete (> 1.4 kbp) 16S rRNA gene fragment was amplified by conventional PCR [6]. For each isolate, the partial 16S rRNA-encoding gene sequence was 100% identical to the type strain of *S. intermedius *(GenBank accession number AF104671). In addition, all three isolates were positive in a PCR assay targeting the *ily *gene, which encodes for *S. intermedius *toxin intermedilysin [7].

## Discussion

The Streptococcus anginosus group (SAG), one of five groups collectively known as viridans group streptococci, consists of the species *S. intermedius, S. anginosus*, and *S. constellatus *[8]. These organisms are commonly associated with purulent infections with abscess formation and less commonly the cause of endocarditis than other viridans streptococci [9-13]. *S. intermedius *has an apparent tropism for the brain and liver, whereas *S. anginosus *and *S. constellatus *have been isolated from a wider range of sites and infections [2,14]. The three cases presented here highlight a common presentation of *S. intermedius *(liver and brain abscesses) and a rare complication (endocarditis).

The identification and classification of streptococci based on hemolytic and Lancefield grouping reactions is cumbersome and lacks discriminatory power. Whiley, et. al. developed phenotypic methods to better differentiate the SAG[14]. Commercially available systems based on these phenotypic tests are widely used and provide a more rapid characterization of each species, but accuracy remains variable [15,16]. This has led to the development of molecular techniques, such as PCR sequencing, to increase the accuracy of identification and clinical profiling [1,7,17,18]. Furthermore, several species-specific genes have been targeted for the development of PCR-based assays for the identification of the SAG organisms [7,18]. Intermedilysin (ILY) is a human-specific cytolysin that directly damages human hepatic cells and is implicated as a potential important virulence factor in causing deep-seated abscesses [19]. The *ily *gene has been shown to be specific for *S. intermedius*. The assay can be used for rapid identification through the use of PCR with the *ily *gene as a species marker gene, which was amplified on all three isolates [7].

Pyogenic liver abscesses are an uncommon, but potentially life-threatening, infection. The first cases of SAG hepatic abscesses were reported in 1975 [20]. Later, a study in 1981 found SAG to be the most common cause of hepatic abscesses [21]. A prospective study by Coreedoira, et. al. compared the incidence and clinical features of SAG liver abscess to liver abscesses caused by other organisms. Members of SAG were most commonly isolated, with *S. intermedius *being the most frequent species. Abscesses also tended to be monomicrobial and the duration of symptoms were longer when compared to other organisms. However, there were no differences in mortality, duration of antibiotics, or complications. The majority of patients required surgical or percutaneous drainage of the abscesses for cure as was the case for our patients, whereas 20% of patients were treated with antimicrobials alone [22].

Members of the SAG are rare causes of endocarditis. Previous studies on infective endocarditis caused by SAG have relied on phenotypic methods for identification. Sussman, et. al. studied 36 patients with viridans streptococcal endocarditis, and identification at the species level was determined by using biochemical tests established by Facklam [23]. Four of the cases were found to be *S. intermedius*. Sixteen isolates from the study were sent to five institutions to confirm identification. Not surprisingly, only 3 were characterized as the same species by all five institutions [24]. Another retrospective study found 29 cases of endocarditis due to SAG, and only 3 were identified as *S. intermedius *[25]. However, the commercial method used in this case, Rapid 32 ID Strep System (bioMérieux), has been shown to be the least accurate in identifying *S. intermedius *[16]. A more recent study by Woo, et. al. applied 16S rRNA sequencing to 6 cases of SAG endocarditis, all of which were identified as *S. anginosus *[3]. To our knowledge, we report the first case of *S. intermedius *endocarditis confirmed by 16S sequence analysis.

Brain abscesses can result via spread from a contiguous focus of infection, hematogenous spread, or as a result from head trauma or neurosurgery [26]. Mortality and morbidity have reduced over the decades in the advent of newer antibiotic therapies and early recognition with CT scans and MRIs [27]. Several case series have defined the clinical characteristics of patients with brain abscesses. All ages are affected with a male predominance. Headache, fever, and mental status changes are the most common initial presenting features, although only one-third of patients have the classic triad. The majority of abscesses are solitary and occur in the frontal lobe. Surgery is required in most patients, and the duration of antibiotics range from 4 to 8 weeks [26-31]. The SAG has been recently recognized as a common cause of brain abscesses, which were collectively identified with other streptococci as viridans group streptococci in prior studies [29,31]. Several retrospective reviews and case reports have identified *S. intermedius *as a significant pathogen among the SAG in the development of brain abscesses [1,2,32,33]. However, no study has compared *S. intermedius *brain abscesses with other members of the SAG or with other bacterial causes.

A unique feature of our cases was the lack of underlying medical problems in our two patients with liver abscesses. Infections caused by SAG are not common in previously healthy individuals. An underlying condition, such as diabetes, cirrhosis, or cancer, is associated with the majority of patients [12,34,35]. Neither of our patients with liver abscesses had chronic illnesses or evidence of immunosuppression. The third case with brain abscess, however, had chronic sinusitis, which is one of the most common predisposing factors [29,31]. The presentation of these cases highlights the range of infection caused by *S. intermedius *that can be encountered in a community setting. Our findings indicate that infections with *S. intermedius *and the other members of the SAG may be more common that previously appreciated.

## Conclusion

*S. intermedius *commonly causes liver and brain abscesses but rarely endocarditis. Our cases demonstrate how *S. intermedius *can cause severe illness in otherwise healthy individuals. Because species in the Streptococcus anginosus group are difficult to identify by routine biochemical tests, newer molecular methods are available to more accurately identify each species. As newer genotyping techniques develop, the clinical characteristics of this diverse group are becoming better understood. These three cases highlight both common and uncommon presentations of *S. intermedius *infection and the utilization of these methods to confirm a rare case of *S. intermedius *endocarditis. Additional studies will be required to more precisely determine the prevalence and range of clinical disease due to infection with the SAG organisms.

## Competing interests

The authors declare that they have no competing interests.

## Authors' contributions

MPT and MCM followed the patients, carried out the molecular studies, and drafted the manuscript. WK supervised all laboratory analyses. VBY participated in the design and coordination of the study and helped draft the manuscript. All authors read and approved the final manuscript.

## Pre-publication history

The pre-publication history for this paper can be accessed here:


